# Organelle Contact-Site Plasticity and Retrograde Stress Codes: Cellular Integration of Biotic–Abiotic Stress in Plants

**DOI:** 10.3390/cells15211974

**Published:** 2026-10-29

**Authors:** Adnan Amin, Wajid Zaman

**Affiliations:** Department of Life Sciences, Yeungnam University, Gyeongsan 38541, Republic of Korea; adnan.amin@yu.ac.kr

**Keywords:** combined stress, membrane contact sites, retrograde signaling, plant immunity, stress memory, ROS, calcium, hormone crosstalk

## Abstract

Plants rarely encounter environmental constraints in isolation. Water deficit, temperature extremes, salinity, flooding, excessive irradiance, nutrient imbalance, herbivory, and microbial infection frequently overlap in time and space, creating composite cellular states that cannot be inferred by adding single-stress responses. This review develops an organelle-centred framework for such integration. We synthesize evidence that the plasma membrane and cell wall initiate partially shared Ca^2+^, reactive oxygen species (ROS), kinase, lipid, and electrical signals, whereas chloroplasts, mitochondria, peroxisomes, the endoplasmic reticulum (ER), and the vacuolar system transform these inputs into compartment-specific retrograde information. We distinguish experimentally supported plant contact-site functions from the hypothesis that contact-site remodeling encodes combined biotic–abiotic stress. We further examine how ABA-, SA-, JA-, and ET-centered hormonal circuits intersect with organellar signals, how the unfolded protein response and selective autophagy set stress thresholds, and how systemic signaling and chromatin-associated memory alter subsequent responses. Because most molecular memory evidence derives from single-stress acclimation or immune priming, persistent memory after combined stress remains an open question. An interface-to-memory model is proposed in which stress combinations are encoded through the timing, amplitude, localization, and persistence of signals, with implications for organelle-resolved experiments and field validation.

## 1. Introduction

Plants experience the environment as a superimposed set of physical, chemical, and biological inputs rather than as discrete treatments. Drought changes leaf temperature, carbon status, and rhizosphere activity; heat modifies membrane properties and immune competence; salinity alters ion balance and water relations; and pathogen infection reprograms host metabolism while itself being shaped by temperature, humidity, and nutrient availability. In crops, water deficit often coincides with high temperature and irradiance; hydroponic polyethylene glycol (PEG) assays isolate osmotic challenge but do not reproduce soil drying, canopy heat load, or rhizosphere change. Ultraviolet-B (UV-B) and excess visible light also alter photosynthetic redox balance. Consequently, the phenotype produced by a stress combination can be synergistic, antagonistic, or qualitatively different from the responses elicited by either component alone. Genetic evidence in Arabidopsis has shown that ABA-dependent abiotic responses can suppress or spatially redistribute immune outputs, with the outcome depending on tissue age and the physiological cost of defence [1]. Broader syntheses likewise indicate that stress integration involves shared receptors, redox and Ca^2+^ signals, transcriptional regulators, and hormone networks, but that their hierarchy is conditional rather than fixed [2,3]. This context dependence is a central reason why tolerance inferred from single-stress assays often loses predictive power when plants are transferred to fluctuating field environments, despite major advances in the molecular description of individual abiotic-stress responses [4].

A useful conceptual shift is therefore to treat the plant cell as an information-processing system in which organelles both execute metabolism and report their functional state. Chloroplasts and mitochondria continuously couple electron transport to redox balance and energy availability, while peroxisomes, the ER, and the vacuole participate in ROS metabolism, lipid exchange, proteostasis, ion storage, and selective degradation. Perturbation of any one compartment can thus create a signal whose meaning depends on the state of the others. Chloroplast-derived ROS and metabolites, for example, can alter nuclear transcription and immunity [5,6], whereas mitochondrial redox status and ATP availability can modulate cellular stress thresholds. The ER is especially positioned to integrate biotic and abiotic inputs because secretory immunity, membrane biogenesis, protein folding, and stress-induced unfolded protein response (UPR) signalling converge in this compartment [7]. The nucleus coordinates transcriptional reprogramming, but organelle-to-nucleus retrograde signals and cytosolic feedback do not form a simple top-down hierarchy. Rather, these processes create a distributed network in which organellar state is repeatedly sampled, compared, and converted into transcriptional and metabolic decisions.

Membrane contact sites (MCSs) add a spatial dimension to this network. An MCS is a domain where two distinct membranes remain closely apposed without fusing; it can organize signaling or facilitate exchange, but proximity alone does not demonstrate direct molecular transfer. Work in eukaryotic systems has established MCSs as organizing centers for lipid homeostasis, Ca^2+^ signaling, organelle dynamics, and metabolic coordination [8]. Plant studies have identified ER contacts with chloroplasts, mitochondria, peroxisomes, endosomes, the vacuole, lipid droplets, and the plasma membrane, and have linked selected interfaces to lipid trafficking and organelle positioning [9,10,11]. ER–plasma membrane contact sites containing plant synaptotagmins and VAP27/NET3C-associated components provide accessible examples of known contact-site architecture [12,13,14]. Recent evidence that chloroplast positioning at the pathogen interface contributes to focal immune responses further suggests that some organelle interfaces are dynamically remodeled during defence [15]. These observations motivate a spatial model of stress integration, while the causal role of MCS remodeling in combined biotic–abiotic stress remains to be tested.

The temporal dimension is equally important. Plant responses are not reset to a naïve state immediately after stress relief. Prior exposure can leave transcriptional, chromatin, protein-conformational, metabolic, and hormonal traces that alter the kinetics of a later response [16,17]. However, most mechanistic evidence for these traces comes from single-stress acclimation or pathogen-induced systemic acquired resistance, and direct evidence for durable memory after combined abiotic–biotic stress remains limited [18]. In *Nicotiana benthamiana*, prior drought acclimation increased subsequent tolerance to *Sclerotinia sclerotiorum* and *Pseudomonas syringae* pv. *tabaci*; this is evidence for sequential cross-protection, not proof that all combined-stress memories persist through recovery [19]. The term “same challenge” therefore requires specification of stress identity, dose, duration, order, developmental stage, and recovery interval. A heat episode followed by pathogen challenge can engage a different cellular network from pathogen attack followed by heat, even when the components are drawn from the same stress pair.

This review examines how plant cells integrate biotic and abiotic stress through a mechanistic continuum that begins at extracellular and membrane interfaces, is transformed through organelle contact sites and retrograde signals, converges on hormone–immune–metabolic control, and can persist as systemic or cellular memory. The specific objectives are to (i) define the shared and stress-selective sensing modules that initiate combinatorial signalling; (ii) evaluate MCSs and organelle-to-nucleus communication as spatial encoders of stress identity; (iii) connect redox, Ca^2+^, proteostasis, autophagy, and hormonal crosstalk to growth–defence decisions; (iv) distinguish short-lived signalling from primed and memory states; and (v) identify experimentally tractable nodes for crop improvement under realistic stress combinations. The resulting framework emphasises testable causal relationships rather than cataloguing stress-responsive genes, and it prioritises cellular interfaces at which information from physically different stresses is most likely to be integrated.

## 2. Stress Perception at the Cell Surface and the First Integration Layer

**Figure 1:**
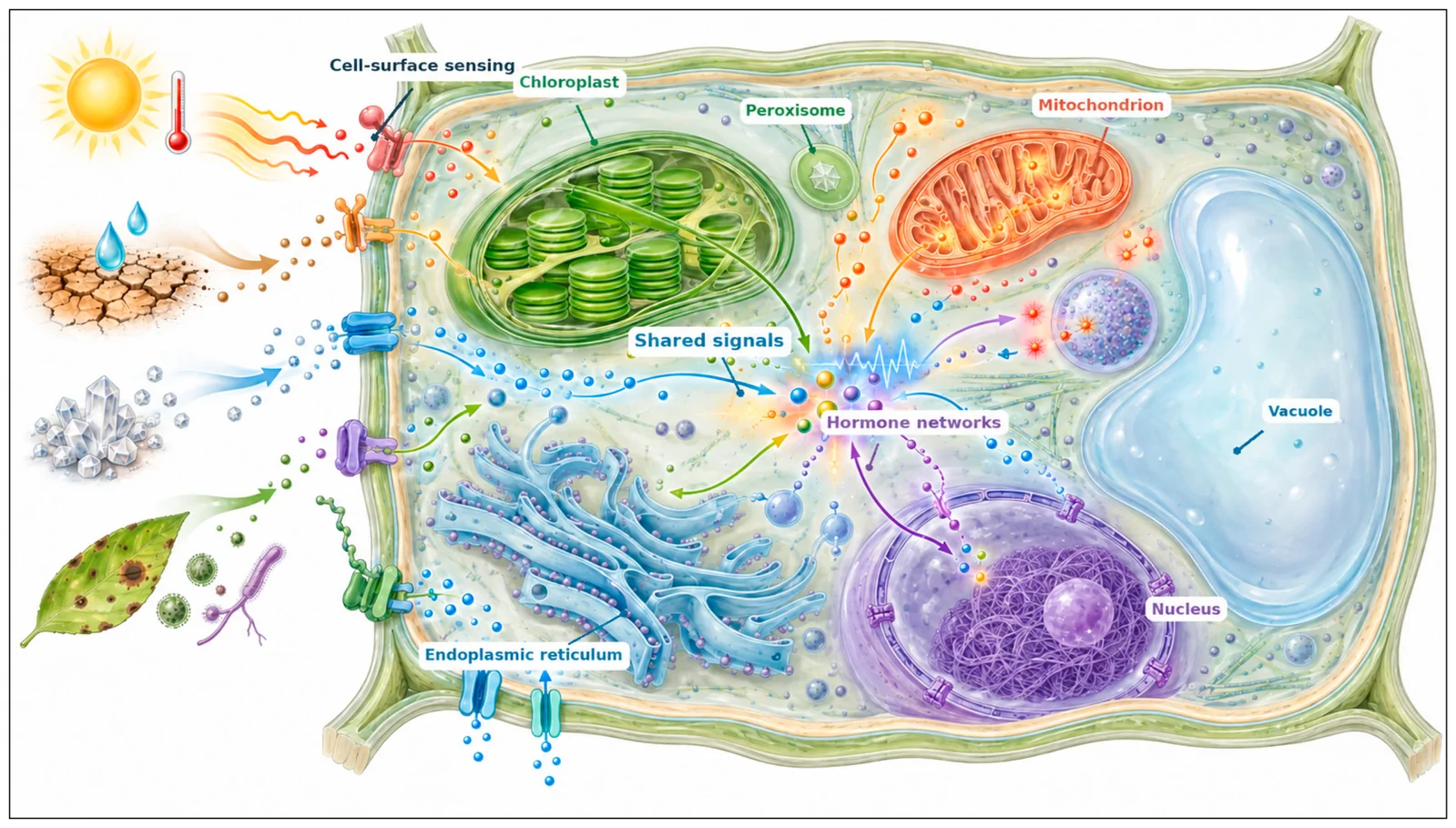
Multiscale integration of biotic and abiotic stress signals in plants. Environmental inputs are perceived through cell-surface, cell-wall, root, ion-channel, and stomatal modules and converted into Ca^2+^, ROS, kinase, redox, lipid, electrical, and nitric-oxide signals. Shared signaling includes ABA-, SA-, JA-, ET-, and growth-hormone networks; these interact with chloroplast, mitochondrial, peroxisomal, ER, vacuolar, and nuclear processes to regulate transcription, metabolism, autophagy, UPR activity, wall reinforcement, defense, acclimation, and systemic resistance. Hormones are shown in the shared-signaling layer rather than as an organelle. The scheme emphasizes convergence and context dependence, not a fixed linear pathway. Abbreviations: ABA, abscisic acid; Ca^2+^, calcium ion; ER, endoplasmic reticulum; ET, ethylene; JA, jasmonic acid; ROS, reactive oxygen species; SA, salicylic acid; UPR, unfolded protein response.

The first integration layer comprises the cell wall, plasma membrane, and apoplast. The apoplast is the extracellular continuum outside the plasma membrane, including the cell-wall matrix and intercellular spaces; the cell wall is the polysaccharide- and protein-rich structural matrix within that continuum. Here, “pathogen” refers to a microbial disease-causing agent; herbivores are treated as a separate class of biotic stress. Root hairs interface with soil water, nutrients, and microbiota, whereas trichomes provide physical and chemical barriers, particularly against insect herbivores [20,21]. Pattern-recognition receptors and receptor-like kinases (RLKs) recognize microbial- or damage-associated molecular patterns, while wall-associated kinases, mechanosensitive channels, osmosensory systems, and membrane lipids report changes in wall integrity, turgor, and membrane state. These sensory classes are not functionally independent. Stress-sensitive biomolecular condensates formed through liquid–liquid phase separation are emerging as non-membrane mechanisms that can alter sensing and response thresholds under dehydration, osmotic stress, and temperature extremes [22]. Cell-wall damage caused by dehydration, salinity, or pathogen enzymes can generate overlapping damage signals, and receptor complexes can share co-receptors, kinases, and downstream Ca^2+^ or ROS modules. Recent analyses of WAK/WAK-like proteins emphasize their dual roles in cell-wall development and stress adaptation, including pectin-associated signalling, ion homeostasis, antioxidant regulation, and immune activation [23]. This shared architecture allows cells to integrate the physical consequences of abiotic stress with microbial perception at an early stage, while ligand specificity and receptor localization preserve sufficient information to avoid a uniform generic response. Figure 1 provides an overview of this hierarchy, placing hormones among shared signaling modules and the nucleus as a transcriptional coordinator within reciprocal organelle–cell networks.

Ca^2+^ is a major second messenger in this early signaling layer because stress perception rapidly changes the activity of plasma-membrane and endomembrane Ca^2+^-permeable channels. The biologically informative variable is not simply Ca^2+^ concentration, but the amplitude, frequency, duration, and subcellular location of the Ca^2+^ transient. These signatures are decoded by calmodulins, calcineurin B-like proteins and their interacting kinases, and calcium-dependent protein kinases (CDPKs), which then phosphorylate ion channels, metabolic enzymes, transcription factors, and ROS-producing NADPH oxidases. K^+^ efflux and anion-channel activity also shape membrane-potential changes and couple ion homeostasis to guard-cell and systemic signaling. Reviews of CDPK function show that individual family members can contribute to both pathogen defense and tolerance to drought, salinity, temperature stress, and oxidative conditions [24,25]. This provides a molecular basis for cross-regulation: a given stress combination can recruit a partially overlapping set of Ca^2+^ sensors while changing their temporal activation. Such temporal multiplexing helps explain why simultaneous and sequential stress exposures can yield distinct outcomes even when they engage many of the same signalling proteins.

ROS production is tightly coupled to Ca^2+^ signalling at the cell surface. Respiratory burst oxidase homologs generate apoplastic ROS, while organelles contribute additional ROS sources whose chemistry and location differ. ROS can oxidise proteins and lipids at damaging concentrations, but at controlled levels they modify cysteine residues, affect phosphatase and kinase activity, alter ion-channel behaviour, and propagate local or systemic information. The integration of ROS and Ca^2+^ signalling is therefore better viewed as a coupled oscillator than as a simple linear cascade. Current models identify reciprocal activation between Ca^2+^-dependent kinases and NADPH oxidases, together with antioxidant systems that shape signal termination and compartment specificity [26,27]. Under combined stress, this network can generate an output that is disproportionately strong or, conversely, buffered if one stress has already induced antioxidant capacity. The same ROS species can therefore represent damage, a second messenger, or a memory-associated priming cue depending on its concentration, source, and timing.

Stomata illustrate how one cellular interface serves abiotic adaptation and immunity. ABA-driven guard-cell closure limits water loss during drought and contributes to responses to salinity, whereas microbial patterns can trigger stomatal immunity. For several bacterial pathogens and some fungi, stomata provide an entry route; entry is not universal, because fungi may penetrate the cuticle or wounds and viruses are commonly introduced by vectors or through damaged tissue [28]. Stomatal aperture also regulates CO_2_ uptake and water-vapor loss, thereby coupling pathogen access to photosynthetic carbon assimilation and transpiration; O_2_ exchange accompanies photosynthetic and respiratory metabolism. Pathogens can manipulate guard-cell behavior, while temperature, CO_2_, humidity, and light alter stomatal physiology and immune signaling. Thus, a change in guard-cell behavior can modify hydraulic status and pathogen access, generating feedback that subsequently changes mesophyll and vascular signaling.

The value of the surface-integration concept is that it distinguishes shared signalling components from shared biological meaning. A Ca^2+^ influx produced by a pathogen elicitor and one produced by osmotic shock may engage related channels but differ in spatial origin, waveform, interacting lipids, and kinase context. The comparison is mechanistically useful, although a pathogen inoculum contains more than a single elicitor: microbial patterns occur alongside enzymes, effectors, toxins, and other metabolites. Osmotic shock is a physical perturbation, whereas field exposure can also alter local microbial communities and tissue chemistry. ROS and electrical signals are likewise interpreted according to their source, timing, and physiological context; K^+^ and anion flux contribute to membrane-potential changes. Table 1 summarises major surface and proximal signaling modules and the information they can contribute under combined stress. The key prediction is that combinatorial specificity emerges from coincidence detection across modules rather than from a dedicated receptor for each stress combination. This prediction can be tested using simultaneous live imaging of Ca^2+^, ROS, membrane potential, and receptor dynamics in defined cell types while varying stress dose, order, and intensity.

## 3. Organelle Contact Sites as Spatial Stress-Integration Platforms

After surface perception, signals are distributed through the cytosol and endomembrane system, where organelle position and membrane topology constrain their propagation. Figure 1 places shared signals and hormone networks between surface sensing and organelle-specific readouts. Chloroplasts, mitochondria, peroxisomes, and the ER are especially sensitive to redox imbalance, altered energy demand, lipid remodeling, and protein-folding stress because these states intersect with their metabolic functions. Stress can also change organelle shape and proximity, modifying opportunities for local signaling. Such geometry may alter signaling efficiency, but colocalization alone cannot establish direct exchange or a causal role in the phenotype. The mechanistic distinction between observed contacts and hypothesized contact-site functions is therefore central to the following sections.

The ER is a particularly extensive contact-forming organelle. It creates interfaces with the plasma membrane, chloroplasts, mitochondria, peroxisomes, endosomes, lipid droplets, and the vacuole, allowing it to couple membrane synthesis and trafficking to Ca^2+^ and lipid homeostasis. In Arabidopsis, the plant cytoskeleton, NET3C, and VAP27 are associated with ER–plasma-membrane contact architecture [12], and ionic stress expands SYT1-enriched ER–PM contacts [13]. A plant ER–mitochondrion contact complex involving TRB1/TRB2 and VAP27-1 has been linked to mitochondrial morphology, energy homeostasis, and mitochondrial-stress-induced mitophagy [11]. SYT1/SYT3 perturbations also compromise plasma-membrane integrity and diacylglycerol homeostasis under several abiotic stresses [14]. These experiments establish stress-responsive ER–PM and ER–mitochondrion contact functions, but do not demonstrate that an MCS encodes a combined biotic–abiotic stress pair. More broadly, plant ER–organelle contacts are dynamic rather than static tether points [10]. The ER also integrates protein-folding status through the UPR, meaning that the same organelle can report both membrane/metabolic imbalance and proteotoxic load. Heat, salinity, pathogen infection, and viral replication can all increase ER demand, through different proximal mechanisms [7]. The resulting ER state can influence secretion of immune proteins, membrane-receptor turnover, autophagy, and nuclear transcription.

Chloroplasts combine photosynthetic electron transport, ROS production, fatty-acid and isoprenoid metabolism, and the biosynthesis of multiple defense-related metabolites. Lipid trafficking between chloroplasts and the ER is important for membrane remodeling during stress [9]. Chloroplast behavior also changes during immunity: epidermal chloroplasts are motile, contain defense components, and reposition in response to fungal penetration attempts [31]. Recent work links chloroplast positioning at pathogen interfaces to focal immune responses [15], supporting active remodeling of organelle proximity at sites of attempted entry. Pathogen-derived proteins can also target chloroplast functions, placing photosynthetic redox and metabolism within the host–pathogen interaction [6]. These observations extend chloroplast biology beyond a simple diffusible retrograde signal, while leaving the transfer route and relevance to specific combined-stress pairs open to experimental testing.

Mitochondria and peroxisomes form another redox-sensitive axis. Mitochondrial electron transport responds rapidly to changes in carbon supply, oxygen availability, and cellular ATP demand, while peroxisomes participate in photorespiration, fatty-acid metabolism, and ROS turnover. Contacts among mitochondria, peroxisomes, chloroplasts, and the ER can support coordinated metabolism and organelle quality control. A recent study showed that mitochondrial ROS can engage a mitochondria-associated ER signaling cascade and prime ER processes for enhanced immunity [32]. This supports interorganellar redox communication; it does not establish that every mitochondrial-to-ER signal is transported directly through a defined contact-site tether or that this pathway has been tested under a biotic–abiotic stress pair. Under heat–pathogen or drought–pathogen conditions, such relay circuits remain plausible determinants of whether metabolic adjustment supports defense or contributes to chronic stress.

**Figure 2:**
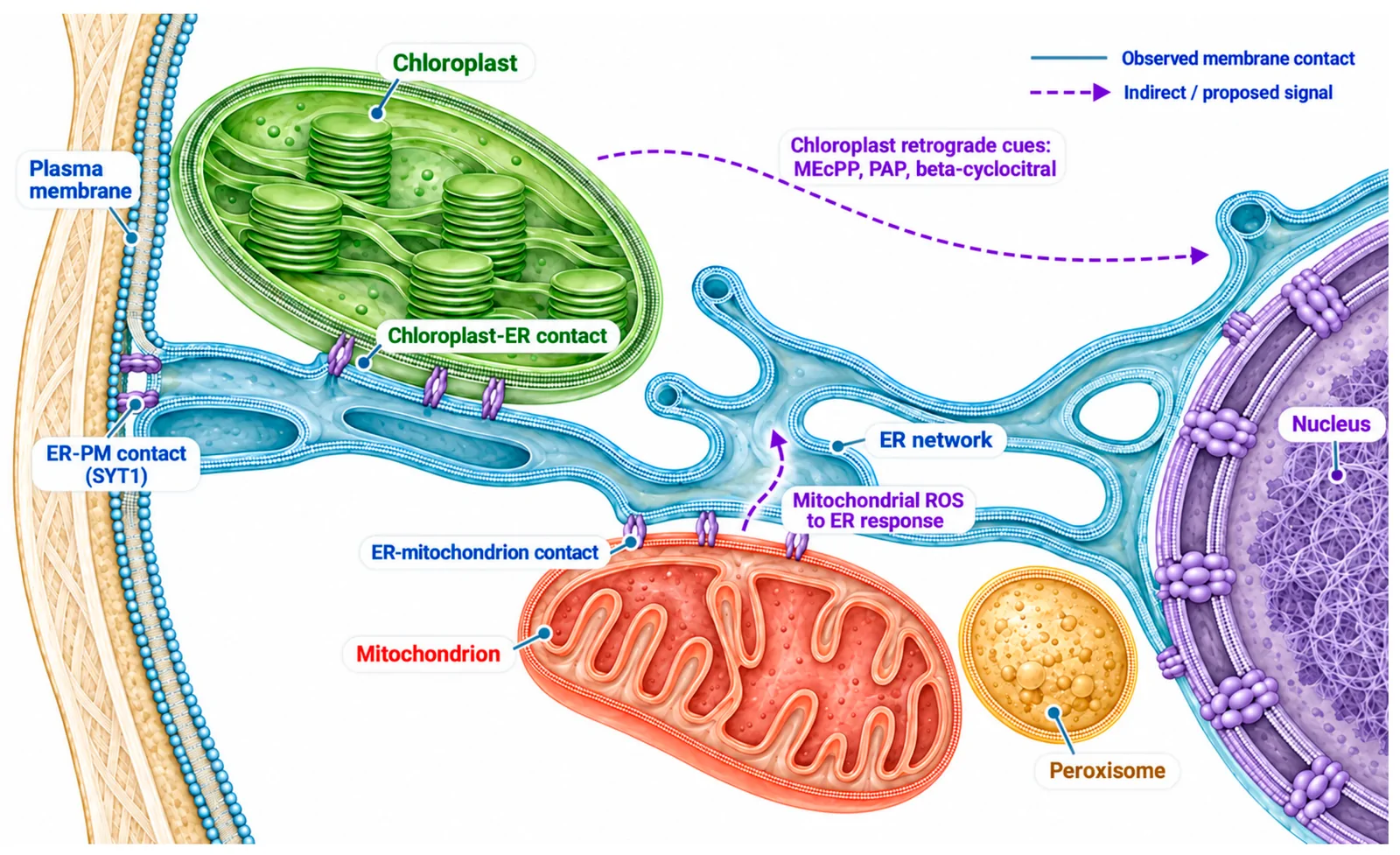
Plant membrane contact sites and retrograde communication. The ER, plasma membrane, chloroplast, mitochondrion, peroxisome, and nucleus are shown as distinct compartments. Solid callout lines identify experimentally observed contact architecture; dashed arrows indicate indirect or proposed signaling routes. The chloroplast-to-nucleus route represents retrograde signaling and is not a physical chloroplast–nucleus contact. Membrane apposition alone does not imply direct molecular transfer. Representative chloroplast retrograde cues include MEcPP, PAP, and β-cyclocitral; mitochondrial ROS can signal to ER-associated processes. Abbreviations: ER, endoplasmic reticulum; MEcPP, methylerythritol cyclodiphosphate; PAP, 3′-phosphoadenosine 5′-phosphate; PM, plasma membrane; ROS, reactive oxygen species; SYT, synaptotagmin.

Figure 2 presents a cell-scale view of established contact architecture and organelle-to-nucleus signaling. Solid callout lines identify experimentally observed membrane appositions; dashed arrows denote indirect or proposed signaling routes, including chloroplast retrograde communication to the nucleus. The chloroplast–nucleus arrow is a signaling relationship, not a physical contact site. The diagram does not assert that molecules cross directly at every contact. Demonstrating functional transfer requires perturbation of a candidate tether or contact geometry together with direct measurement of lipid, ion, redox, or signaling flux. Live-cell imaging, proximity labeling, and genetically encoded sensors make these tests increasingly feasible.

The MCS framework generates testable predictions for combined-stress biology, but stress-pair-specific contact-site encoding remains a hypothesis. In plants, stress-responsive ER–PM contact remodeling and chloroplast repositioning at pathogen interfaces have experimental support [13,14,15]; causal evidence that a specific MCS configuration encodes a simultaneous biotic–abiotic stress combination is not yet established. If such encoding occurs, different stress pairs should remodel distinct contact subsets rather than causing a uniform increase in proximity. Contact remodeling should precede at least some transcriptional changes, and perturbing a relevant tether should alter the interaction between stresses more strongly than either single stress. These predictions should be tested across epidermal, guard, mesophyll, vascular, and root cells, with stress dose and duration controlled. This approach moves the field beyond descriptive colocalization while keeping the current evidence and the proposed model distinct.

## 4. Retrograde Stress Codes: From Organelle State to Nuclear Reprogramming

Retrograde signaling describes the transfer of information from organelles to the nucleus and cytosol so that gene expression and metabolism can be adjusted to organelle state. In chloroplasts, candidate signals include ROS, redox changes, tetrapyrrole-related intermediates, 3′-phosphoadenosine 5′-phosphate (PAP), methylerythritol cyclodiphosphate (MEcPP), β-cyclocitral, and other reactive electrophile or lipid-derived products. Contemporary work emphasizes that photosynthetic ROS are not a single message; singlet oxygen, superoxide-derived products, hydrogen peroxide, and lipid oxidation products arise in different locations and under different electron-transport states, creating distinguishable outputs [33]. Chloroplast redox signaling is integral to immunity because infection modifies photosynthesis, antioxidant capacity, and defense-hormone biosynthesis [5,6]. These chloroplast-to-nucleus routes are biochemical retrograde pathways, not evidence of a physical chloroplast–nucleus MCS. Under combined stress, the nucleus therefore receives an organelle-state vector whose interpretation depends on concurrent cytosolic kinase and hormone activity.

Mitochondrial retrograde information similarly couples respiratory state to stress adaptation. Changes in electron transport, ROS production, redox couples, organic-acid metabolism, and ATP availability can influence nuclear gene expression and interact with chloroplast-derived signals. Many core subunits of chloroplast photosystems and mitochondrial electron-transport complexes are encoded by organellar genomes, whereas catalases and many antioxidant enzymes are nuclear-encoded and targeted to specific compartments. Glutathione (GSH) is present in multiple cellular compartments, but its synthesis, transport, and redox state are not equivalent across those compartments; whole-tissue measurements can therefore obscure organelle-specific redox status. In chloroplasts, photosynthetic electron transport also changes ferredoxin and NADPH reduction, coupling energy capture to reductive capacity and ROS signaling. Because photosynthesis and respiration are metabolically interdependent, chloroplasts, mitochondria, peroxisomes, and the ER can generate compensatory or reinforcing signals while each also influences the nucleus [34]. Such interorganellar coupling is especially relevant when one stress perturbs energy supply, and another increases the energetic cost of defense.

The ER contributes a different class of retrograde information centered on proteostasis and membrane status. Accumulation of unfolded proteins activates UPR pathways that increase folding capacity, ER-associated degradation, and other homeostatic functions [35]. Because secreted receptors, defense proteins, and membrane transporters pass through the secretory system, ER stress can directly alter the cell’s ability to sustain immune and abiotic-stress responses. Viral replication can also exploit or burden ER membranes, creating a direct intersection between biotic stress and proteostasis [7]. Thus, the ER can convert apparently unrelated stresses into a common variable: the ratio between folding/secretory demand and processing capacity. When this ratio exceeds a threshold, the cell may reprioritize translation, membrane trafficking, autophagy, and growth, effectively changing the gain of other stress-response pathways.

ROS and Ca^2+^ provide a bridge between organellar retrograde signals and rapid cytosolic signaling. Their interaction is bidirectional and compartmentalized. Ca^2+^ uptake and release can alter organelle metabolism and ROS production, whereas redox changes regulate channels, pumps, kinases, and phosphatases. Integrative reviews identify molecular nodes that connect these pathways under abiotic stress [27], while whole-plant imaging demonstrates that electrical, Ca^2+^, ROS, and hydraulic signals can be coordinated during systemic responses [30]. These observations argue against treating retrograde signaling as a slow transcription-only process. Instead, organelle state can influence rapid electrical and Ca^2+^ events, which then feed back on organelle function. Combined stress may therefore be encoded across multiple timescales: milliseconds to minutes in ion and redox dynamics, minutes to hours in protein modification and transcription, and days in chromatin and metabolic memory.

A stress code becomes mechanistically meaningful only if downstream readers can discriminate among signal combinations. Candidate readers include redox-sensitive transcription factors, MAPKs, CDPKs, bZIP factors activated by ER stress, and hormone-regulated transcriptional modules. Specificity may arise from coincidence: a transcription factor could require both phosphorylation and a redox-dependent change in localization; a promoter could respond only when a chloroplast signal coincides with SA accumulation; or a UPR branch could alter translation so that an otherwise identical immune signal produces a different protein output. Table 2 organizes major organelle-derived signals according to their likely sources, proximal readouts, and unresolved questions under combined stress. The table also highlights a recurring gap: many signals are well established under single stress, but the rules by which their relative timing and amplitude encode stress combinations remain incompletely tested.

The practical consequence is that future experiments should preserve temporal and spatial information rather than relying only on end-point transcriptomes. Two stress treatments can yield similar sets of differentially expressed genes while arriving there through distinct sequences of Ca^2+^, ROS, kinase, and organelle-state changes. Conversely, early signalling can differ substantially yet converge on similar terminal expression because buffering and feedback restore homeostasis. Live reporters for organelle redox state, ATP, pH, Ca^2+^, membrane potential, and UPR activity, combined with perturbation of contact-site proteins, can reveal the hidden trajectories that precede transcriptomic outcomes. This trajectory-centred approach is likely to be particularly informative for identifying the mechanistic basis of non-additive combined-stress phenotypes.

## 5. Hormone–Immune–Metabolic Crosstalk and Growth–Defence Decisions

Hormones convert cellular stress information into coordinated changes in physiology and development. ABA is central to water-deficit and osmotic responses; SA is strongly associated with resistance to many biotrophic pathogens and systemic acquired resistance; JA and ET are prominent in wound, herbivore, and necrotroph responses; while auxin, cytokinins, brassinosteroids, and gibberellins couple growth to stress adaptation. These pathways are interconnected rather than arranged in a fixed hierarchy. Under combined stress, ABA can antagonize or reconfigure SA-associated immunity, but the direction and magnitude depend on tissue, timing, genotype, and pathogen lifestyle [1,2]. Growth hormones further alter resource allocation and developmental sensitivity, making the network a state-dependent control system for immediate defence and future reproductive cost. Hormones therefore modify how Ca^2+^, ROS, kinase, and organellar signals are translated into gene expression and physiology, consistent with current models of hormone-regulated abiotic stress adaptation [29].

The ABA–SA relationship illustrates why fixed labels such as antagonistic or synergistic can mislead. In *Arabidopsis thaliana*, moderate drought increased susceptibility to *Pseudomonas syringae* pv. tomato DC3000 while suppressing the CBP60g/SARD1 defence axis [36]. By contrast, prior drought acclimation in *Nicotiana benthamiana* reduced subsequent disease from *P. syringae* pv. *tabaci* and *Sclerotinia sclerotiorum* [19]. These contrasting outcomes show that plant species, pathogen lifestyle, drought dose, and exposure order matter. Elevated temperature also weakens Arabidopsis resistance to *P. syringae* through loss of ICS1-mediated salicylic-acid biosynthesis and increased bacterial effector translocation [37]. Thus, a rise in ABA or temperature-sensitive immune change can be protective, neutral, or detrimental depending on when it occurs relative to pathogen entry and immune activation; neither outcome supports a universal ABA-to-SA inhibitory rule.

JA and ET provide a second integration axis. Wounding and herbivory induce JA biosynthesis, while ET modulates defense and senescence and interacts with JA-responsive transcription factors. JA signaling also intersects with ABA during dehydration and with growth pathways through JAZ repressors and MYC transcription factors. Recent network-oriented syntheses emphasize that JA crosstalk is implemented through transcription-factor interactions, targeted protein degradation, and post-translational control rather than by hormone concentration alone [38]. In *Arabidopsis*, combined heat and osmotic stress altered subsequent resistance to *Pseudomonas syringae* and *Botrytis cinerea*; the heat–osmotic combination increased susceptibility to *B. cinerea* and reduced expression of PDF1.3, BOS1, and THI2.2 [39]. This result links a defined combined abiotic treatment to pathogen-specific defense outcomes without implying that JA–ET interactions have the same sign across pathogens or species. Temperature-dependent modulation of immune cell biology further illustrates how abiotic conditions reshape defense circuitry [40].

N-hydroxypipecolic acid (NHP) and the regulatory protein NONEXPRESSOR OF PATHOGENESIS-RELATED GENES 1 (NPR1) connect local immune perception to systemic preparedness. Exogenous or infection-induced NHP can drive a systemic transcriptional program and prime SA-associated defenses in an NPR1-dependent manner [41]. This pathway illustrates how a mobile metabolite changes the response threshold of distal tissue rather than simply turning defense on continuously. In a combined-stress context, the effectiveness of this primed state depends on the metabolic and redox capacity of recipient cells, as well as on environmental conditions that influence stomata, photosynthesis, or hormone balance. The broader implication is that systemic immunity is superimposed on local organellar states that can amplify or constrain the response. Stress order is therefore critical: an abiotic event occurring during the establishment or maintenance of systemic acquired resistance (SAR) can reshape later pathogen resistance even if the stresses are not simultaneous.

**Figure 3:**
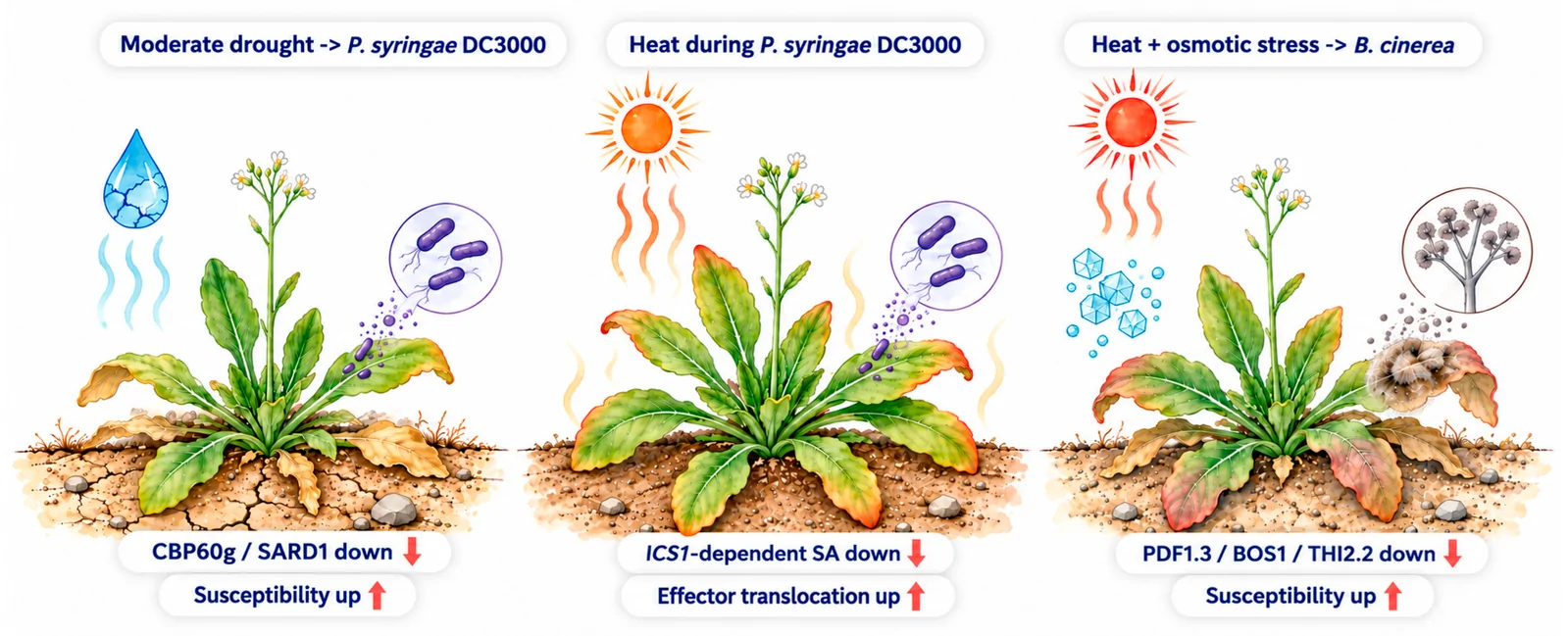
Experimental examples of context-dependent biotic–abiotic interactions in Arabidopsis. Moderate drought followed by *Pseudomonas syringae* pv. tomato DC3000 challenge suppresses CBP60g/SARD1 and increases susceptibility; elevated temperature during P. syringae challenge reduces ICS1-dependent salicylic acid production and promotes effector translocation; combined heat and osmotic stress before Botrytis cinerea challenge increases susceptibility and reduces PDF1.3, BOS1, and THI2.2 expression. These outcomes are specific to the tested plant, pathogen, dose, and sequence and should not be generalized to all hormone–pathogen combinations. Abbreviations: BOS1, BOTRYTIS SUSCEPTIBLE 1; CBP60g, calmodulin-binding protein 60g; ICS1, ISOCHORISMATE SYNTHASE 1; PDF1.3, PLANT DEFENSIN 1.3; SARD1, SAR DEFICIENT 1; SA, salicylic acid; THI2.2, THIONIN 2.2.

Metabolism supplies both signals and constraints to hormonal crosstalk. Photosynthetic source activity determines carbon and energy supply, while sink demand from expanding leaves, roots, reproductive tissues, and developing seeds competes for assimilates; phloem transport, mineral status, and pathogen load further shift this balance. Stomatal closure and heat can reduce carbon assimilation at the same time that defense raises ATP and biosynthetic demand. Chloroplasts provide precursors for JA and other defense-related metabolites, while mitochondrial and peroxisomal metabolism influence redox balance and energy. Growth restraint can conserve resources during severe stress, but chronic suppression may reduce yield or alter food composition even if survival improves. Figure 3 summarizes three experimentally studied stress combinations with pathogen-specific outcomes rather than a universal hormone-interaction map. Resilience assessment should therefore include yield stability and, where relevant, nutritional quality and mineral content such as iron and zinc [42].

This network perspective also clarifies why engineering a single hormone pathway can produce unintended trade-offs. Constitutive activation of defense may impose growth penalties, while maximizing ABA-mediated water conservation may alter carbon gain, stomatal immunity, or pathogen microenvironments. A more promising strategy is to manipulate conditional nodes that preserve dynamic range—for example, tissue-specific receptors, stress-inducible transcription factors, kinase docking interactions, or priming mechanisms that enhance response speed without maintaining high defense constitutively. Costs may differ between a localized response and systemic acquired resistance because distal tissues maintain a primed state while continuing growth and reproduction. These approaches require phenotyping under combinations and sequences of stress, with both disease control and carbon-use costs measured rather than evaluating single-stress tolerance separately.

## 6. Proteostasis, the UPR, Selective Autophagy, and Cellular Quality Control

Combined stress imposes a proteostasis problem in addition to a signalling problem. Heat destabilises protein folding, salinity and dehydration alter ionic and osmotic conditions, oxidative stress damages proteins and membranes, and infection increases the demand for synthesis, processing, and secretion of defence proteins. The ER senses one major component of this burden through the UPR. Plant UPR branches increase chaperone and folding capacity, adjust ER-associated degradation, and reduce the load of proteins entering an already stressed secretory system [35]. Because plasma-membrane receptors, cell-wall proteins, and secreted defense components depend on ER function, proteostasis directly constrains signaling competence. The ER can therefore determine whether upstream immune and abiotic-stress signals are successfully executed or converted into chronic stress and cell death.

Autophagy provides a complementary mechanism by removing damaged proteins, aggregates, and organelles and by recycling metabolites when energy or nutrients are limiting. Selective autophagy allows this process to be targeted through receptors that connect specific cargo to ATG8-family proteins. Autophagy occurs in the cytoplasm through autophagosome formation and delivery to the vacuole; its cargo routes are compartment-specific rather than a single proteolytic system shared by all organelles. Chloroplasts contain internal Clp, FtsH, and Deg proteases and can be removed through chlorophagy; chloroplast-associated protein degradation (CHLORAD) uses cytosolic ubiquitination and the cytosolic 26S proteasome for selected envelope proteins [43]. Plant selective autophagy has established roles in biotic and abiotic stress responses [44]. NBR1 is particularly relevant because it participates in removal of stress-induced protein aggregates and has been linked to heat-stress memory, virus interactions, pexophagy, ABA-related processes, and other stress responses [45]. These functions position selective autophagy at the intersection of proteotoxic stress, organelle quality control, hormone signaling, and memory.

ER-phagy further integrates the UPR with selective degradation. When ER stress becomes severe or prolonged, portions of the ER can be removed through autophagic pathways, helping restore ER architecture and function [46]. This creates a thresholded response: moderate stress may be resolved by chaperones and ER-associated degradation, whereas persistent damage recruits larger-scale membrane turnover. Under combined stress, one component may precondition this threshold. A prior heat episode could increase chaperone capacity and buffer a later infection-associated secretory load; alternatively, sustained heat could exhaust proteostasis and make the same infection more damaging. These are testable alternatives rather than established combined-stress memory mechanisms, reinforcing the need to measure stress history and recovery directly.

Organelle quality control also affects signal fidelity. Damaged chloroplasts or mitochondria can become excessive ROS sources that blur the distinction between regulated signaling and oxidative injury. Selective removal, repair, and antioxidant buffering therefore set the usable dynamic range of redox signals. Mitochondrial ROS-mediated communication with the ER provides an example of a redox cue priming another compartment rather than merely triggering damage [32]. Chloroplasts use internal proteases and CHLORAD for protein quality control, while whole-organelle removal engages autophagic routes; the cytosolic 26S proteasome is not a plastid stromal proteasome [43]. Similar quality-control logic applies to mitochondrial and peroxisomal turnover. Table 3 summarizes mechanisms that may determine whether combined stress produces acclimation, chronic defense, or cell death, while emphasizing that their causal roles in stress-pair outcomes remain unevenly tested.

Proteostasis thus functions as a cellular budget for stress signaling. A plant may possess intact receptors and transcription factors yet fail to express resilience if secretory, folding, repair, and degradation systems cannot support the required protein flux. Crop improvement may therefore benefit from improving the capacity and recoverability of cellular execution systems rather than maximizing defence amplitude. Conditional enhancement of UPR buffering, selective autophagy, or organelle quality control is a candidate strategy, but broader tolerance across stress combinations has not been established; excessive UPR or autophagy can also promote growth inhibition or cell death.

## 7. Systemic Signaling, Stress Memory, and Priming Across Recurrent Challenges

Local stress responses become whole-plant responses through vascular, electrical, hydraulic, ROS, Ca^2+^, hormone, and metabolite signals. Whole-plant imaging has shown that membrane-potential, Ca^2+^, ROS, and hydraulic changes can be coordinated during responses to wounding or high light, with glutamate receptor-like proteins and plasmodesmatal function contributing to signal propagation [30]. Such rapid signaling can prepare tissues that have not yet encountered the stress, changing their physiological state before local exposure. This matters under combined stress because a root-derived water-deficit signal can reach leaves before a foliar pathogen challenge, while local infection can alter systemic water relations or stomatal behavior. A distant leaf therefore integrates incoming systemic information with its own organellar and developmental state.

Priming describes a state in which prior exposure changes the responsiveness of a subsequent reaction. Proposed memory mechanisms include persistent transcription-factor occupancy, chromatin marks, altered DNA methylation, histone modification, small RNAs, protein phosphorylation, metabolite pools, and changes in hormone sensitivity [16,17,18]. These mechanisms differ in persistence: some support short-term acclimation and decay during recovery, whereas others can remain detectable longer under specific genotypes and developmental conditions. Much of this mechanistic evidence comes from single-stress experiments; systemic acquired resistance is a well-characterized immune-priming example, but it is not equivalent to demonstrated memory after combined abiotic–biotic stress. Cross-protection after drought acclimation has been reported [19], yet durable memory after complete recovery and re-challenge with a combined stress remains insufficiently established. A primed plant may appear recovered while retaining a lower activation threshold, but persistence must be measured rather than inferred from an early response.

Immune priming provides a well-defined example. NHP accumulation after local microbial attack contributes to systemic acquired resistance and sensitizes distant tissue for stronger defense-gene induction [41]. Beneficial root-associated microbes can establish induced systemic resistance through JA/ET-centered and other signaling routes, changing aboveground defense readiness without constitutively activating all defenses. These pathways are external modifiers of the organelle-centered processes reviewed here: drought, salinity, temperature, infection, and agronomic inputs can alter root exudation, microbial composition, nutrient status, and host hormone balance. Their effects may therefore contribute to cross-protection or cross-susceptibility, but should not be treated as evidence for a defined organelle-contact mechanism.

**Figure 4:**
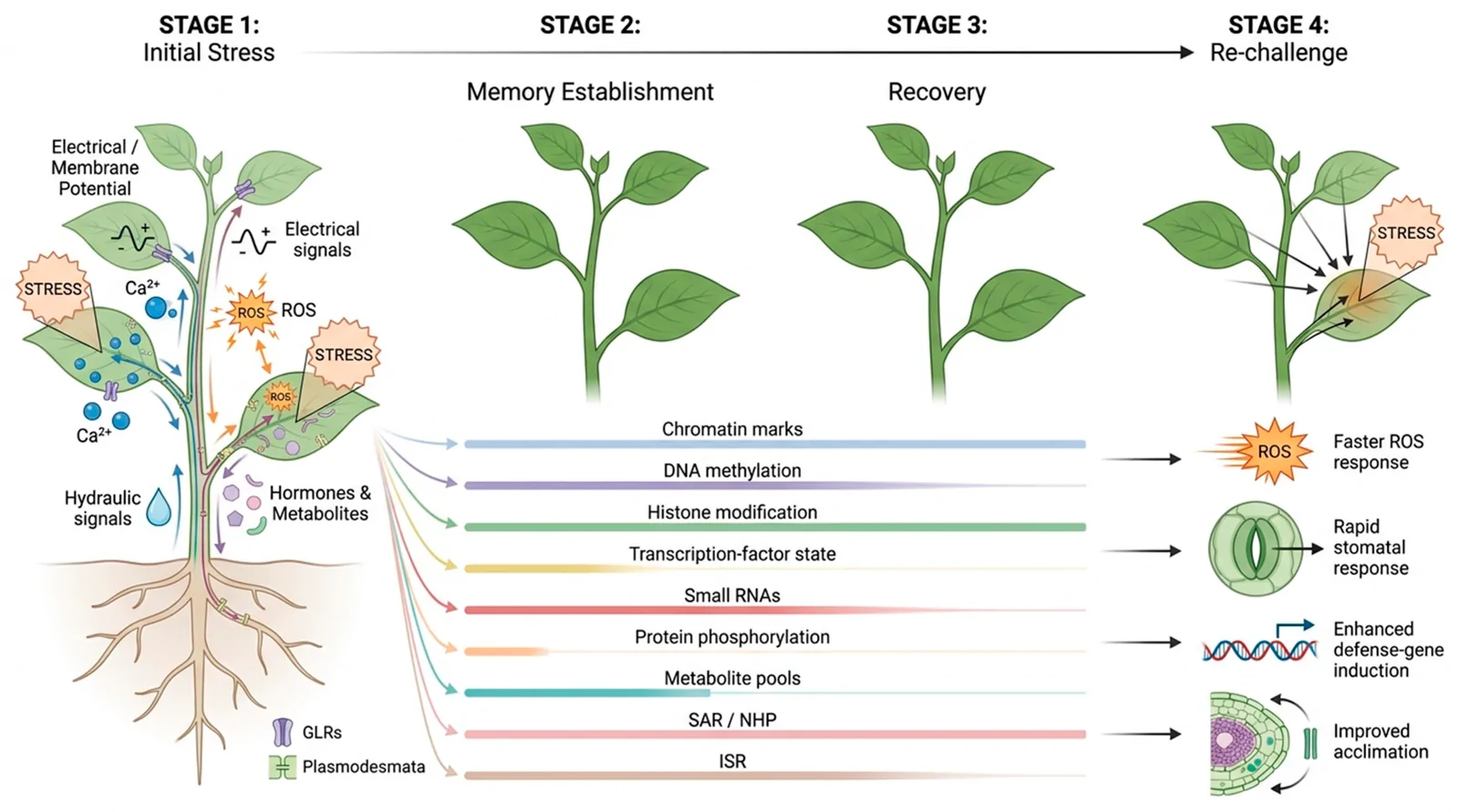
Multilayer stress memory and priming across recovery and re-challenge. Prior stress can leave changes in chromatin, DNA methylation, histone acetylation, transcription-factor state, small RNAs, phosphorylation, metabolites, and systemic immune signals. During re-challenge, these states may alter response thresholds and kinetics, producing faster ROS, stomatal, transcriptional, or acclimatory outputs. Persistence differs among molecular layers; many mechanisms shown are established mainly in single-stress acclimation or immune priming and should not be read as validated combined-stress memory.

Figure 4 integrates these ideas into a four-stage sequence of initial stress, memory establishment, recovery, and re-challenge. The depicted chromatin, methylation, histone, transcription-factor, small-RNA, phosphorylation, metabolite, SAR/NHP, and ISR layers are not assumed to change in parallel or to be established together under combined stress. The figure emphasizes that molecular traces can decay at different rates and may converge on faster ROS, stomatal, transcriptional, or acclimatory responses during re-challenge. Recent work on crop stress memory underscores both the potential value and uncertainty of translating priming from controlled environments to agriculture [48,49]. Memory duration, genotype, developmental stage, stress dose, and the match or mismatch between priming and challenge all influence the outcome.

A rigorous combined-stress memory experiment should separate simultaneous interaction from sequential memory. At minimum, designs should include every single stress, the simultaneous combination, both stress orders, recovery controls, and re-challenge groups. Physiological phenotyping should be linked to time-resolved molecular measurements so persistent memory marks can be distinguished from residual stress. Recovery duration, stress dose, and developmental stage should be reported, and candidate chromatin, NHP/NPR1, autophagy, guard-cell, and contact-site changes should be measured in parallel with organelle-level reporters. These designs can identify nodes at which prior experience changes the sign or amplitude of a later response without assuming that any one mechanism persists beyond the initial stimulus.

## 8. From Mechanism to Resilient Crops: Systems Biology and Translation

Mechanistic understanding of combined stress will require data that preserve cell identity, time, and environmental sequence. Bulk transcriptomics has been indispensable for defining stress-responsive pathways, but it averages across cells that can experience different pathogen loads, water status, or organelle states. RNA-seq is not inherently nuclear-only: organellar transcripts can be recovered when library preparation retains them and the reference genome includes chloroplast and mitochondrial sequences; analyses mapped only to a nuclear reference can omit these reads. Organelle-aware mapping should therefore be paired with compartment-specific reporters, targeted RNA or protein measurements, and spatial information. Single-cell and spatial approaches can resolve heterogeneity; cell-type-specific and spatially organized defense responses are detectable at single-cell resolution [50,51,52], and crop-focused syntheses highlight the feasibility of extending these approaches beyond model species [53]. For combined stress, these methods should be coupled to pathogen location, stomatal state, vascular position, and organelle reporters so molecular changes can be assigned to defined physiological contexts.

Multi-omics integration is the second requirement. Transcript abundance alone cannot resolve protein phosphorylation, metabolite priming, membrane lipid remodeling, chromatin state, or compartment-specific redox dynamics. Genomics, transcriptomics, proteomics, phosphoproteomics, metabolomics, lipidomics, epigenomics, ionomics, and phenomics each capture different levels of the stress system [54]. Genomic and transcriptomic approaches remain important for connecting natural variation and stress-responsive regulatory networks to breeding targets [55]. The analytical objective should be causal network reconstruction. Candidate hubs are most convincing when supported by multiple evidence classes—for example, a kinase whose phosphorylation changes before transcription, whose perturbation alters a contact-site or organelle signal, and whose effect is strongest under a stress combination. Network models should incorporate directionality and time delay because a highly connected late response may be a consequence rather than a driver of integration.

Genome editing can then be used to test and manipulate these causal nodes. CRISPR/Cas systems, base editing, prime editing, and multiplex approaches provide routes to alter receptor domains, kinase motifs, transcription-factor binding sites, hormone sensitivity, or contact-site tethers with greater precision than constitutive overexpression [56]. The highest-value targets may be regulatory regions or protein interfaces that tune response thresholds while preserving essential growth functions. Multiplex editing is particularly attractive for combined stress because redundancy and compensatory signaling are common; however, it also increases the risk of hidden pleiotropy. Edited lines should therefore be evaluated across stress sequences, developmental stages, and microbiome contexts rather than only under a single acute treatment.

The microbiome is considered here as an external modifier of plant stress integration rather than a primary mechanistic subject. Root exudates shape microbial recruitment, while beneficial microbes can influence nutrient acquisition, hormone balance, antioxidant capacity, and systemic resistance [57]. Infection, drought, salinity, and management inputs such as fertilization or pesticide exposure can alter exudation and reshape rhizosphere communities; those changes can in turn affect root architecture, ion homeostasis, and defense readiness. A genotype selected in sterile or simplified conditions may therefore behave differently in soil. Microbiome engineering is best evaluated as a complementary intervention alongside host genetics, using defined communities and measured plant outcomes rather than assuming that microbial effects are uniform.

Field validation remains essential because environmental heterogeneity introduces fluctuating temperature, vapor-pressure deficit, irradiance, UV-B exposure, soil moisture, pathogen pressure, and microbial communities that can change within hours or across the canopy. Local plants may experience rapid transitions from hot, dry days to cool or freezing conditions, while drought and heat frequently overlap. A mechanism robust in growth chambers must retain its benefit when stress dose and order are uncontrolled. High-throughput phenotyping, environmental sensors, remote imaging, electrical measurements, volatile monitoring, and repeated molecular sampling can connect laboratory-defined signaling hubs to field performance [58]. Visible injury, including scorch, chlorosis, and premature senescence, should be evaluated alongside yield stability, disease burden, recovery, resource-use efficiency, and nutritional quality. For common bean, drought and heat can affect mineral composition and dietary value as well as productivity [42]. Genotype-by-environment analysis can determine whether a candidate provides broad resilience or transfers vulnerability from one stress to another.

**Figure 5:**
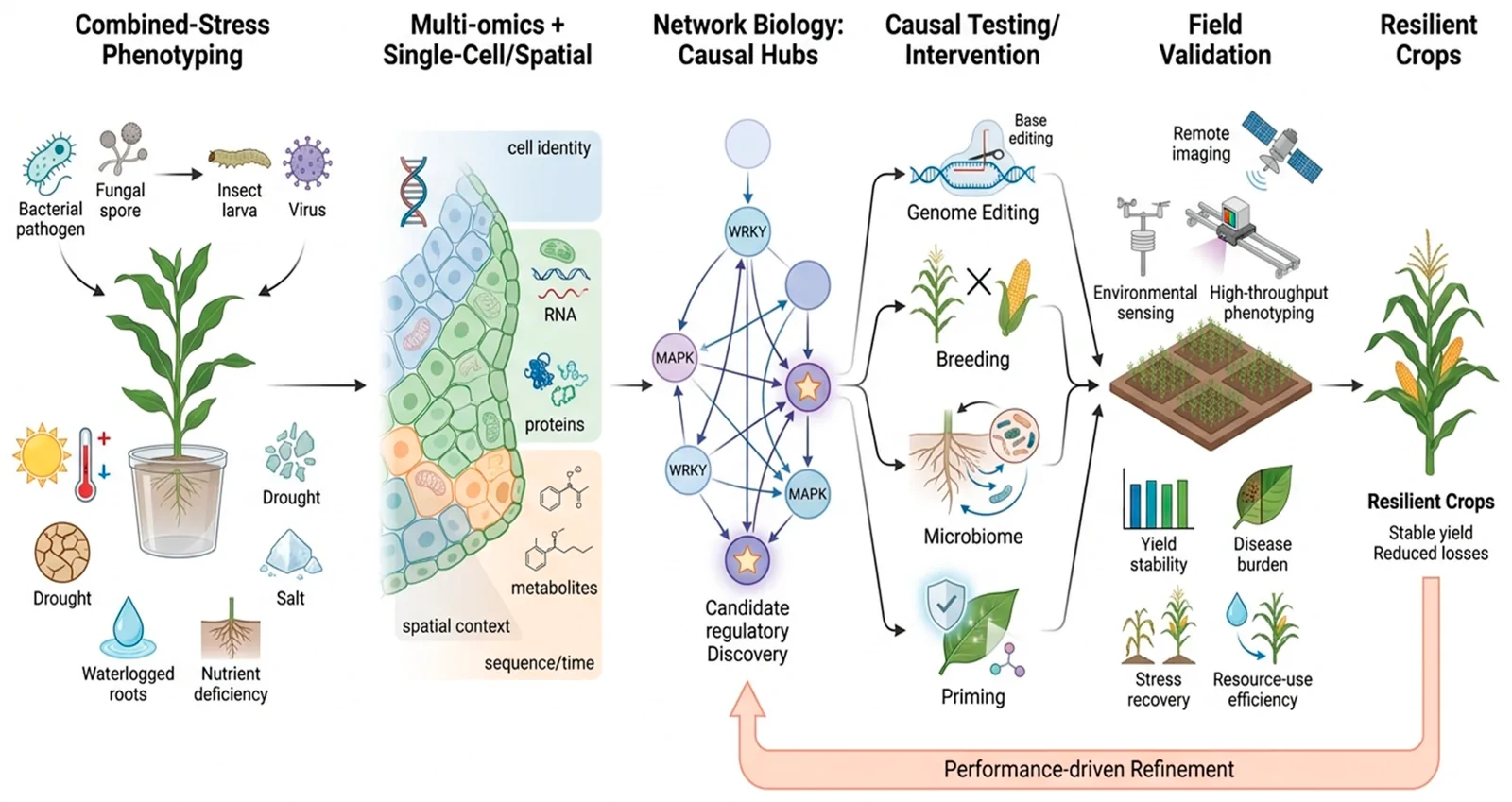
Systems workflow for translating combined-stress mechanisms into resilient crops. Realistic stress phenotyping is integrated with multi-omics, single-cell/spatial analysis, and network biology to identify candidate regulatory hubs. Causal testing through genome editing, breeding, microbiome engineering, and priming is followed by field validation for resilience, yield stability, disease burden, nutritional quality where relevant, and loss reduction. Field outcomes feed back to discovery so environmental, developmental, or cellular variables can refine the mechanistic model.

Figure 5 summarizes a translational workflow from combined-stress phenotyping through multi-omics, single-cell/spatial analysis, network inference, candidate-hub validation, genome editing, breeding, microbiome engineering, priming, and field testing. Field assessment should include both agronomic performance and, where relevant, nutritional quality. The feedback arrow is essential: field failures should be used to revise the mechanistic model and identify missing environmental or cellular variables. This iterative strategy is better suited to complex stress biology than a one-directional pipeline in which a gene identified under one laboratory stress is moved directly into breeding. Recent syntheses of abiotic-stress mechanisms and climate-sensitive immunity similarly emphasize the need to connect molecular understanding to realistic environmental validation [59,60].

Host-directed priming and breeding remain complementary routes for testing stress resilience. Induced systemic resistance and plant-growth-promoting bacteria can modify defense readiness and abiotic tolerance [61,62], but their benefits should be assessed under representative soil, climate, and pathogen conditions. These strategies can be integrated with cell-wall, autophagy, and specialized-metabolism research [63,64] while maintaining the review’s focus on organelle-centered integration.

## 9. Conclusions and Perspectives

The central challenge in combined-stress biology is not a shortage of stress-responsive genes, but an incomplete understanding of how cells assign meaning to overlapping signals. The evidence reviewed here supports an organelle-centered model in which stress identity is encoded by the timing, location, amplitude, and persistence of signals across the cell surface, endomembrane system, and metabolic organelles. MCSs provide spatially restricted opportunities for exchange; retrograde pathways report organelle state; hormone and kinase networks adjust response gain; and proteostasis determines whether signaling can be executed without loss of cellular integrity. This framework explains why similar components can generate different outcomes under different stress combinations while distinguishing established mechanisms from proposed integration models.

A major unresolved question is whether particular contact-site configurations constitute reproducible stress states. Plant studies have identified ER–PM tethers, stress-responsive contact remodeling, and chloroplast positioning at pathogen interfaces. However, causal evidence that a defined MCS configuration encodes a simultaneous biotic–abiotic stress combination remains limited. Establishing this will require quantitative imaging, candidate-tether perturbation, and simultaneous measurement of transferred signals and plant outcomes. The current evidence makes these tests tractable but does not yet validate contact-site engineering as a crop-improvement strategy.

A second priority is to connect cellular trajectories with memory. The same stress pair may produce different outcomes depending on whether organelle quality control, chromatin state, hormone sensitivity, or systemic priming has been altered by prior experience. Experiments that vary stress dose, order, and recovery are essential for distinguishing co-occurrence from historical contingency. Live reporters and single-cell/spatial methods can assign persistent changes to specific cell populations rather than inferring them from whole-organ averages. Durable memory after combined biotic–abiotic stress should be treated as an experimental question, not an assumed property of priming. For crop improvement, contact-site components and other integration nodes remain research candidates rather than validated field targets. More direct routes for current testing include environment-matched priming, genetic variation, breeding, and stress-sequence phenotyping, with field validation required for each proposed benefit. Resilience assessment should preserve growth and reproductive performance while measuring recovery, disease, yield stability, and nutritional quality where relevant. An interface-to-memory framework can guide this work by identifying where diverse environmental signals may converge, while avoiding the assumption that a mechanistic candidate will improve crops before it has been tested in realistic conditions.

## Figures and Tables

**Table 1:** Surface and proximal signalling modules that integrate biotic and abiotic stress information.

Integration Module	Representative Components	Information Encoded	Combined-Stress Implication	Key Refs
Cell-wall surveillance	WAK/WAKL, pectin-associated receptors, wall integrity sensors	Wall damage, mechanical strain, pectin status	Abiotic wall deformation and pathogen-derived damage can converge before cytosolic signalling	[23]
Pattern recognition	PRRs/RLKs, co-receptors, immune kinases	MAMP/DAMP identity and receptor complex state	Abiotic conditions can alter receptor trafficking, membrane state, and downstream immune gain	[2,28]
Ca^2+^ signatures	Channels, CDPKs, CBL-CIPKs, calmodulins	Amplitude, frequency, duration, compartment	Stress combinations can reuse Ca^2+^ machinery while changing waveform and decoding	[25,27]
Apoplastic ROS	RBOHs, peroxidases, antioxidant systems	Local redox state and propagation potential	Can amplify Ca^2+^ signaling or become damaging when organellar ROS is already high	[26,27]
Stomatal interface	ABA receptors, ion channels, ROS/Ca^2+^, immune receptors	Hydraulic demand, pathogen entry, atmospheric conditions	A single guard-cell decision changes both water loss and susceptibility to entry	[28,29]
Electrical/systemic signalling	GLRs, plasmodesmata, membrane potential changes	Rapid tissue-to-tissue alert	Links local wounding/high-light or pathogen-related signals to distal acclimation	[30]

Abbreviations: ABA, abscisic acid; CBL-CIPK, calcineurin B-like protein–CBL-interacting protein kinase; CDPK, calcium-dependent protein kinase; DAMP, damage-associated molecular pattern; GLR, glutamate receptor-like protein; MAMP, microbe-associated molecular pattern; PRR, pattern-recognition receptor; RBOH, respiratory burst oxidase homolog; RLK, receptor-like kinase; ROS, reactive oxygen species; WAK/WAKL, wall-associated kinase/wall-associated kinase-like protein; Ca^2+^, calcium ion.

**Table 2:** Organelle-derived and contact-site-associated information streams relevant to combined stress.

Organelle/Interface	Representative Stress-Derived Information	Primary Cellular Readouts	Why it Matters Under Combined Stress	Key Refs
Chloroplast	Photosynthetic ROS, redox state, β-cyclocitral, PAP, MEcPP, lipid-derived signals	Nuclear transcription, antioxidant remodelling, defence metabolism	Photosynthesis and immunity compete for and share redox/metabolic capacity	[5,6,33]
Mitochondrion	Respiratory ROS, ATP/ADP status, redox and organic-acid state	Energy allocation, redox signalling, nuclear responses	Can relay metabolic stress to ER and alter immune readiness	[26,32]
ER	Unfolded proteins, membrane/lipid imbalance, secretory load	UPR, ERAD, translation/secretory adjustment	Biotic and abiotic stresses converge on a finite folding and secretion capacity	[7,35]
Peroxisome	H_2_O_2_/redox state, photorespiratory and lipid metabolism	Antioxidant balance, metabolic exchange	Couples photosynthetic state to ROS buffering and organelle quality control	[5,10]
ER–chloroplast contact	Lipid exchange, proximity-dependent metabolic coupling	Membrane remodelling, organelle positioning	May tune chloroplast membrane repair and retrograde output during stress	[9,10]
Chloroplast–pathogen interface	Focal organelle positioning and contact remodelling	Localised immune output	Provides spatial mechanism for concentrating defence at attempted entry sites	[15,31]
ER–mitochondrion axis	Ca^2+^, lipid/redox coupling, stress relay	Proteostasis and bioenergetic coordination	Mitochondrial ROS can prime ER-associated immune processes	[10,11,32]

Abbreviations: ATP, adenosine triphosphate; ADP, adenosine diphosphate; ER, endoplasmic reticulum; ERAD, ER-associated degradation; H_2_O_2_, hydrogen peroxide; MEcPP, methylerythritol cyclodiphosphate; PAP, 3′-phosphoadenosine 5′-phosphate; ROS, reactive oxygen species; UPR, unfolded protein response.

**Table 3:** Proteostasis and quality-control systems that determine combined-stress capacity.

Quality-Control Layer	Core Function	Stress-Combination Role	Potential Intervention	Key Refs
UPR	Increase folding and ER quality-control capacity	Buffers secretory/proteotoxic load imposed by heat, salt and infection	Stress-inducible tuning of UPR branches	[7,35]
ER-associated degradation	Remove misfolded ER proteins	Prevents chronic ER stress that would compromise receptor/defense protein processing	Conditional enhancement of ERQC capacity	[35]
ER-phagy	Selective turnover of ER domains	Resets ER architecture after prolonged or severe stress	Tune receptors controlling ER turnover rather than constitutive autophagy	[46]
NBR1-mediated selective autophagy	Remove aggregates and selected cargo	Links heat memory, virus interactions, ABA-related responses and organelle quality control	Modify cargo recognition or stress-dependent NBR1 activity	[45]
Organelle autophagy/quality control	Remove damaged chloroplast/mitochondrial/peroxisomal material	Prevents uncontrolled ROS while maintaining signaling range	Tissue-specific modulation of organelle turnover	[44,47]
Proteasome–autophagy coordination	Balance soluble protein degradation with bulk/selective recycling	Determines whether defense is sustainable or transitions to senescence/cell death	Target stress-induced adaptors and degradation thresholds	[44]

Abbreviations: ABA, abscisic acid; ER, endoplasmic reticulum; ERAD, ER-associated degradation; ER-phagy, autophagic turnover of ER; NBR1, neighbor of BRCA1 gene 1; QC, quality control; ROS, reactive oxygen species; UPR, unfolded protein response.

## Data Availability

No new data were created or analyzed in this study. Data sharing is not applicable to this article.
